# cDNA synthesis for *BCR-*ABL1 detection at the MMR level: the importance of using the appropriate kit

**DOI:** 10.1186/s12575-015-0014-x

**Published:** 2015-02-09

**Authors:** Jianxiang Chi, Chryso Pierides, Andrie Mitsidou, Andri Miltiadou, Petroula Gerasimou, Paul Costeas

**Affiliations:** The Center for the Study of Haematological Malignancies, Nicosia, Cyprus; Karaiskakio Foundation, Nicosia, Cyprus

**Keywords:** BCR-ABL, cDNA synthesis, CML, MMR, MRD

## Abstract

**Background:**

The synthesis of complementary DNA (cDNA) for use in the detection of *BCR-ABL1* at the Major Molecular Response (MMR) level is a well-established method used by clinical laboratories world-wide. However, the quality of cDNA provides sensitivity challenges and consequently affects the detection of Minimal Residual Disease (MRD).

**Results:**

Herein, we evaluated six commercially available kits for the synthesis of cDNA according to amplification success rate, linearity and *ABL1* copy number. Based on our results, the Invitrogen SuperScript® III Reverse Transcriptase kit performed better, among the ones used in this study, for the cDNA synthesis, followed by the First Strand cDNA Synthesis Kit for RT-PCR (AMV), available from Roche Applied Sciences.

**Conclusions:**

Accurate and sensitive testing for the detection of abnormal transcripts, allows the correct stratification and treatment of patients. Hence, the use of a suitable kit for the cDNA synthesis is of great importance. This study provides a comprehensive point of reference for clinical laboratories in an attempt to optimize *BCR-ABL1* detection. We propose that the Invitrogen SuperScript® III Reverse Transcriptase kit is the most suitable, among the ones used in this study, for the cDNA synthesis to be used for the detection of *BCR-ABL1* at the MMR level in a CML MRD assay.

**Electronic supplementary material:**

The online version of this article (doi:10.1186/s12575-015-0014-x) contains supplementary material, which is available to authorized users.

## Background

Chronic myelogenous leukemia (CML) is a myeloproliferative disorder that accounts for about 20 percent of newly diagnosed leukemia cases in adults [1,2], with median age of 60 to 65 years [3]. It is characterized by a large accumulation of mature myeloid cells [4] and most CML patients demonstrate *BCL-ABL1* fusion genes in hematopoietic progenitor cells, resulting from a reciprocal translocation between chromosomes 9 and 22, leading to a shortened chromosome 22, the Philadelphia (Ph) chromosome [2,5]. In CML, the Ph chromosome gives rise to *BCR-ABL1* and its active protein tyrosine kinase product p210^BCR-ABL^ [5-7]. Recurrence or long term persistence of *BCR-ABL1* after bone marrow transplantation is indicative of disease relapse [8,9]. Following allogeneic stem cell transplantation (ASCT), the Ph chromosome may not be detectable, although this does not exclude the presence of residual leukemic cells [10]. Due to the very low levels of residual *BCR-ABL1* transcripts present in the blood of patients, its detection is achieved by Real Time Quantitative Polymerase Chain Reaction (RQ-PCR), which detects abnormal transcripts with a sensitivity of 1 in 10^4^ or 1 in 10^5^ cells [11]. Minimal Residual Disease (MRD) is monitored by RQ-PCR for the detection of *BCR-ABL*1 leukemia-specific targets, indicating patients with highest risk of relapse [12]. Such quantitation also allows for correct stratification of patients and predicts the duration of disease remission [13,14]. It is widely considered that a major molecular response (MMR) is the goal of therapy, as it is associated with a favorable progression-free survival [15]. Hence, the achievement of a MMR, particularly within the first year of therapy, is predictive of a strong cytogenetic remission [16].

According to the European LeukemiaNet (ELN), RQ-PCR assessment of *BCR-ABL1* transcript levels must be carried out every 3 months until a MMR has been achieved and then repeated at least every 6 months [17,18]. Results should be expressed as a ratio of *BCR-ABL1* to *ABL1* (or other housekeeping genes) x 100%; converted to the international scale, the ratio ≤ 0.1% defined MMR [17]. *BCR-ABL1* transcript levels ≤ 10% at 3 months, < 1% at 6 months, and ≤ 0.1% from 12 months onward are set to define optimal response, whereas > 10% at 6 months and > 1% from 12 months onward indicate failure, and call for a change of treatment (second-line therapy) [18]. In the case of second-line therapy, *BCR-ABL1* transcript levels ≤ 10% at 3 and 6 months, < 1% at 12 months and ≤ 0.1% from 12 months onward are set to define an optimal response, whereas > 10% at 6 and 12 months indicate failure [18].

Existing guidelines, define an optimum response to therapy by the attainment of MMR by 18 months of the start of therapy [11,19], with current methodology expressing transcript numbers per microgram of leukocyte RNA [20] or as a ratio of *BCR-ABL1/ABL1* on a log scale [21,22]. International Randomized Study of Interferon and STI571 (IRIS) study laboratories have established an international reporting scale for *BCL-ABL1* quantitation by RQ-PCR defining an MMR [13,15]. “MR4.5 is defined as a *BCR-ABL1* ratio lower than 0.0032% on the international scale (IS) provided copy numbers for the control gene are at least 32 000 (i.e. a 4.5-log reduction)” [23].

Currently, clinical laboratories world-wide are using different commercially available kits, based on different primers and enzymes, for the synthesis of cDNA. This can potentially influence the results and consequently the detection of MRD. Yet, no study is available comparing these kits in order to define whether these are suitable for the detection of *BCR-ABL1* at the MMR level. In this study, we compare six commercially available cDNA synthesis kits (Table 1), for the identification of the most suitable kit for use in a CML MRD assay. For each kit, the manufacturers’ protocols are used with *BCR-ABL1/ABL1* samples at 0.32%, 0.032% and 0.0032%, followed by the Europe Against Cancer (EAC) protocol for the detection of *BCR-ABL1* and *ABL1* gene using the TaqMan® method. The results are analysed and scored according to failure rate, linearity and *ABL1* copy number.

**Table 1:** **Total score for the six commercially available kits, based on three parameters: a) success rate score, b)** ***ABL1*** **score and c) linearity**

**Product name**	**Success rate score**	***ABL1*** **score**	**Linearity score**	**Total score**
AffinityScript Multiple Temperature cDNA Synthesis Kit	2	3	6	11
**(Product number: 200436, Agilent Technologies)**				
iScript™ Select cDNA Synthesis Kit	1	1	2	4
**(Product Number: 170–8897, Bio-Rad)**				
SuperScript® III Reverse Transcriptase	6	6	6	18
**(Product Number: 18080–044, Invitrogen)**				
QuantiTect® Reverse Transcription Kit	5	2	4	11
**(Product Number: 205311, Qiagen)**				
First Strand cDNA Synthesis Kit for RT-PCR (AMV)	5	5	4	14
**(Product Number: 11 483 188 001, Roche Applied Sciences)**				
Transcriptor First Strand cDNA Synthesis Kit	5	4	2	11
**(Product Number: 04 379 012 001, Roche Applied Sciences)**				

## Results and discussion

Serial measurement of leukemia-specific transcripts is a valuable approach for monitoring individual patients and in some cases for indicating the need to reassess therapy [22]. Following the high efficacy of the standard CML treatment, the need for an accurate method for monitoring the response is existent. Accurate quantification of *BCR-ABL1* transcripts has been proven to be the most sensitive available method with a great prognostic impact and serving for an early assessment of MRD [12]. Hence, identification of which commercially available kit is more suitable for the synthesis of cDNA is essential, in order to achieve better results at the MMR level. Herein, the six kits tested were classified based on three parameters: a) success rate, b) *ABL1* copy number and c) linearity.

At first, a success rate analysis was performed, where the reverse transcription enzyme was considered successful if it amplified the *BCR* from the cDNA. The success rate was calculated based on the number of wells that were successfully amplified divided by the total well number for BCR amplification, with the enzyme with the highest success rate achieving the highest score (with the highest score being 6 and the lowest 1). All kits detected 0.32% *BCR-ABL1* copy numbers. Using the SuperScript® III Reverse Transcriptase kit the *BCR* was successfully amplified from the cDNA at all levels, achieving a score of 6 (18/18). QuantiTect® Reverse Transcription, First Strand cDNA Synthesis kit for RT-PCR and the Transcriptor First Strand cDNA Synthesis kit achieved 15 successful reads at the 0.0032% level, attaining a score of 5 (15/18). AffinityScript Multiple Temperature cDNA Synthesis kit achieved 13 successful reads at the 0.0032% level, attaining a score of 2 (13/18) and the iScript™ Select cDNA Synthesis kit recorded 5 successful reads at the 0.032% level and one successful read at the 0.0032% level (12/18), attaining the lowest score of 1 (Additional file 1: Table S1).

In theory, amplification of the minimal number of *BCR* is more likely with higher *ABL1* copy numbers. Consequently, the total *ABL1* copy number was used as the validation factor. The sum of the *ABL1* copy numbers observed at 0.32%, 0.032% and 0.0032% was calculated and a score of 1 to 6 was assigned to the individual kits, with the highest score being assigned to the kit with the highest copy number, in this case SuperScript® III Reverse Transcriptase (Additional file 1: Table S2).

According to linearity, the ratio of *BCR/ABL1* at 0.32% copy numbers is expected to be 10 times more than the ratio of *BCR/ABL1* at 0.032% copy numbers. The same also applies for 0.032% versus 0.0032%. In this study, absolute values were calculated using the formulae: *ratio of* 0.032% − (*ratio of* 0.32% *divided by* 10) and *ratio of* 0.0032% − (*ratio of* 0.032% *divided by* 10). Finally, a score of 1 to 6 was given to each kit based on the absolute values observed. Smaller absolute values were given higher scores, with the highest being a score of 6, assigned to the kit with the highest final score after adding the two individual scores. The highest ratios were observed by the SuperScript® III Reverse Transcriptase and AffinityScript Multiple Temperature cDNA Synthesis kit, both achieving a score of 6. Whereas the iScript™ Select cDNA Synthesis kit and the Transcriptor First Strand cDNA Synthesis kit achieved the lowest scores (Additional file 1: Table S3).

In order to acquire a total score for each kit, the sum of the success rate, *ABL1* and linearity scores was observed. Using this strategy, all three parameters were taken into consideration for the evaluation of the different cDNA synthesis kits. Based on our findings, the kit with the highest acquired score was the SuperScript® III Reverse Transcriptase, by Invitrogen, achieving an overall score of 18, followed by the First Strand cDNA Synthesis Kit for RT-PCR, by Roche, with an overall score of 14 (Table 1).

## Conclusion

The detection of *BCR-ABL1* at the MMR level after bone marrow transplantation, which is indicative of disease recurrence, depends on the quality of the cDNA. Accurate detection of *BCR-ABL1* transcripts is also vital for the line of treatment to be followed. Hence, it is important for a clinical laboratory to perform accurate and sensitive testing for the detection of abnormal transcripts, allowing the correct stratification, as well as the right line of treatment for each patient. In this study, we evaluated the six aforementioned commercially available kits for the synthesis of cDNA according to failure rate, linearity and *ABL1* copy number. All three parameters were taken into account in a scoring system and the kit with the highest overall final score was chosen. Based on our results, we are proposing that the SuperScript® III Reverse Transcriptase kit, by Invitrogen, is the more suitable, among the ones used in this study, for cDNA synthesis to be used for the detection of *BCR-ABL1* at the MMR level in a CML MRD assay. From this essential comparison of the available kits, it should be obvious that the quality of the cDNA is of great importance and a key element for the correct management of the patient. Our analysis provides a comprehensive point of reference for clinical laboratories in an attempt to optimize *BCR-ABL1* detection. Our analysis method is also applicable for the assessment of other commercially available kits and methods.

## Materials and methods

### Samples

Bone marrow or peripheral blood samples were obtained from healthy individuals and Ph-positive CML patients, referred to our laboratory for screening. Three samples found positive for t(9;22) with *BCR-ABL1* copy number around 100 – 300 and RNA concentration above 100 ng/μl were used. For each positive sample, the *BCR-ABL1* copy number was adjusted to 0.32%, 0.032% and 0.0032%. The RNA concentration of all samples was adjusted to 100 ng/μl. Negative samples were pooled together and the *ABL1* copy number was measured.

### RNA extraction and cDNA synthesis

Total RNAs were extracted from bone marrow or peripheral blood from healthy individuals and Ph-positive CML patients using the QIAamp® RNA Blood Mini Kit (QIAGEN) on the QiaCube automated platform (QIAGEN). cDNA was synthesized following the manufacturers’ protocols. The amount of input RNA used was 10 μg and the final volume for all reactions was adjusted to 50 μl with ddH_2_O. cDNA was stored at - 30°C overnight and then used for RQ- PCR.

### Real-time quantitative polymerase chain reaction

A standardized protocol established by the EAC program was used for the quantitation of *BCR-ABL1* [24]. 5 μl cDNA were amplified in 25 μl reaction volume with 12.5 μl TaqMan® Universal Master Mix and 1 μl primer per probe mix. Amplification was performed over 50 cycles. Reactions were carried out using serial dilutions for both fusion and control genes. All RQ-PCR reactions were performed in duplicate on a 7500 ABI platform (Applied Biosystems®).

## Additional file

Additional file 1: Table S1. Success rates of the six commercially available kits for 0.32%, 0.032% and 0.0032% *BCR-ABL* copy numbers. **Table S2.** *ABL* copy number for the six commercially available kits for 0.32%, 0.032% and 0.0032% ratios. **Table S3.** Absolute values of the six commercially available kits, as calculated from the ratios of 0.32%, 0.032% and 0.0032%.

## Abbreviations

*ABL1*: Abelson
ASCT: Allogeneic stem cell transplantation
*BCR*: Breakpoint cluster region
cDNA: Complementary deoxyribonucleic acid
CML: Chronic myelogenous leukemia
EAC: Europe against cancer
ELN: European LeukemiaNet
IRIS: International randomized study of interferon and STI571
IS: International scale
MMR: Major molecular response
MR: Molecular response
MRD: Minimal residual disease
Ph: Philadelphia chromosome
RNA: Ribonucleic acid
RQ-PCR: Real-time quantitative polymerase chain reaction
RT-PCR: Reverse transcriptase polymerase chain reaction
