# Supplementary material for: cDNA synthesis for BCR-ABL1 detection at the MMR level: the importance of using the appropriate kit
Source: Biol Proced Online. 2015 Feb 9;17(1):4. doi: 10.1186/s12575-015-0014-x (PMC4321704; doi:10.1186/s12575-015-0014-x)
Supplement: Additional file 1: Table S1. — Success rates of the six commercially available kits for 0.32%, 0.032% and 0.0032% BCR-ABL copy numbers. Table S2. ABL copy number for the six commercially available kits for 0.32%, 0.032% and 0.0032% ratios. Table S3. Absolute values of the six commercially available kits, as calculated from the ratios of 0.32%, 0.032% and 0.0032%. [file 12575_2015_14_MOESM1_ESM.docx]

**cDNA synthesis for *BCR-*ABL1 detection at the MMR level: The importance of using the appropriate kit for complementary DNA synthesis**

**Authors:** Jianxiang Chi*^,1^, Chryso Pierides*^,1^, Andri Mitsidou^1,^ Andrie Miltiadou^2^, Petroula Gerasimou^2^ and Paul Costeas^1,2^.

1. The Center for the Study of Haematological Malignancies, Nicosia, Cyprus.
2. Karaiskakio Foundation, Nicosia, Cyprus.

**TABLE OF CONTENTS**

**Table S1.** Success rates of the six commercially available kits for 0.32 %, 0.032 % and 0.0032 % *BCR-ABL* copy numbers.

**Table S2.** *ABL* copy number for the six commercially available kits for 0.32 %, 0.032 % and 0.0032 % ratios.

**Table S3.** Absolute values of the six commercially available kits, as calculated from the ratios of 0.32 %, 0.032 % and 0.0032 %.

**Table S1. Success rates of the six commercially available kits for 0.32 %, 0.032 % and 0.0032 % *BCR-ABL* copy numbers.**

| **Product Name** | **0.32 %** | **0.032 %** | **0.0032 %** | **Total** | **Score** |
| --- | --- | --- | --- | --- | --- |
| AffinityScript Multiple Temperature cDNA Synthesis Kit | 6/6 | 6/6 | 5/6 | 17/18 | 2 |
| iScript™ Select cDNA Synthesis Kit | 6/6 | 5/6 | 1/6 | 12/18 | 1 |
| SuperScript® III Reverse Transcriptase | 6/6 | 6/6 | 6/6 | 18/18 | 6 |
| QuantiTect® Reverse Transcription Kit | 6/6 | 6/6 | 3/6 | 15/18 | 5 |
| First Strand cDNA Synthesis Kit for RT-PCR (AMV) | 6/6 | 6/6 | 3/6 | 15/18 | 5 |
| Transcriptor First Strand cDNA Synthesis Kit | 6/6 | 6/6 | 3/6 | 15/18 | 5 |

**Table S2. *ABL* copy number for the six commercially available kits for 0.32 %, 0.032 % and 0.0032 % ratios.**

| **Product Name** | **0.32 %** | **0.032 %** | **0.0032 %** | **Total** | **Score** |
| --- | --- | --- | --- | --- | --- |
| AffinityScript Multiple Temperature cDNA Synthesis Kit | 28400.00 | 22166.67 | 21500.00 | 72066.67 | 3 |
| iScript™ Select cDNA Synthesis Kit | 21200.00 | 20766.67 | 15033.33 | 57000.00 | 1 |
| SuperScript® III Reverse Transcriptase | 64566.67 | 58000 | 63533.33 | 186100.00 | 6 |
| QuantiTect® Reverse Transcription Kit | 13316.00 | 21966.67 | 28675.00 | 63957.67 | 2 |
| First Strand cDNA Synthesis Kit for RT-PCR (AMV) | 32900.00 | 38900.00 | 38700.00 | 110500.00 | 5 |
| Transcriptor First Strand cDNA Synthesis Kit | 25300.00 | 25000.00 | 24166.67 | 74466.67 | 4 |

**Table S3. Absolute values of the six commercially available kits, as calculated from the ratios of 0.32 %, 0.032 % and 0.0032 %.**

| **Product Name** | **0.32 %** | **0.032 %** | **0.0032 %** | **Absolute value of 0.32 % to 0.032 %** | **Score of 0.32 % vs 0.032 %** | **Absolute value of 0.032 % to 0.0032 %** | **Score of 0.032 % vs 0.0032 %** | **Overall Score** |
| --- | --- | --- | --- | --- | --- | --- | --- | --- |
| AffinityScript | 0.329 | 0.085 | 0.007 | 0.052 | 2 | 0.0019 | 6 | 6 |
| iScript™ Select | 0.314 | 0.037 | 0.020 | 0.006 | 5 | 0.0164 | 1 | 2 |
| SuperScript® III | 0.248 | 0.029 | 0.010 | 0.004 | 6 | 0.0075 | 2 | 6 |
| QuantiTect® | 0.344 | 0.056 | 0.010 | 0.021 | 3 | 0.0040 | 4 | 4 |
| First Strand | 0.300 | 0.046 | 0.012 | 0.016 | 4 | 0.0070 | 3 | 4 |
| Transcriptor First Strand | 0.256 | 0.078 | 0.011 | 0.053 | 1 | 0.0033 | 5 | 2 |
